# Interplay between metabotropic glutamate type 4 and adenosine type 1 receptors modulate synaptic transmission in the cerebellar cortex

**DOI:** 10.3389/fphar.2024.1406238

**Published:** 2024-08-15

**Authors:** Simon Bossi, Hervé Daniel, Heather McLean

**Affiliations:** Institut des Neurosciences (NeuroPSI) UMR9197 CNRS, Université Paris-Saclay, Saclay, France

**Keywords:** cerebellum, mGlu4 receptor, adenosine type-1 receptor, interaction, neurotransmission

## Abstract

The synapses between parallel fibers and Purkinje cells play a pivotal role in cerebellar function. They are intricately governed by a variety of presynaptic receptors, notably by type 4 metabotropic glutamate (mGlu4) receptors and type 1 adenosine (A1) receptors both of which curtail glutamate release upon activation. Despite their pivotal role in regulating synaptic transmission within the cerebellar cortex, functional interactions between mGlu4 and A1 receptors have remained relatively unexplored. To bridge this gap, our study delves into how mGlu4 receptor activity influences A1 receptor-mediated alterations in excitatory transmission. Employing a combination of whole-cell patch clamp recordings of Purkinje cells and parallel fiber presynaptic fluorometric calcium measurements in acute rat and mouse cerebellar cortical slices, our results reveal functional interactions between these receptor types. These findings hold implications for understanding potential roles of these presynaptic receptors in neuroprotection during pathophysiological conditions characterized by elevated glutamate and adenosine levels.

## 1 Introduction

The cerebellum, long recognized for its role in motor control and learning (De Zeeuw and Ten Brinke, 2015) has more recently been linked to a wide range of cognitive and emotional functions (Schmahmann and Sherman, 1998; Sacchetti et al., 2009; Strata et al., 2011; Fastenrath et al., 2022; Ciapponi et al., 2023). At the heart of cerebellar function are the Purkinje cells (PC), the principal output neurons of the cerebellar cortex. These GABAergic neurons project primarily to the deep cerebellar nuclei and receive glutamatergic input from climbing fibers originating in the inferior olive and parallel fibers (PF) arising from cerebellar granule cells. Excess synaptic glutamate, resulting from an ischemic insult for example, can have important consequences on cerebellar function. PCs are extremely vulnerable to ischemia (Brasko et al., 1995; Ardeshiri et al., 2006) and respond to the ensuing excess extracellular glutamate by a strong anoxic depolarizing current (Hamann et al., 2005; Mohr et al., 2010). Several lines of evidence suggest that metabotropic glutamate type 4 (mGlu4) receptors play a neuroprotective role by regulating synaptic glutamate levels in pathophysiological scenarios such as epileptic seizures (Meldrum et al., 1999) or ischemic insults (Rossi et al., 2000; Panatier et al., 2004; Kalda et al., 2006; Domin et al., 2016). At the PF-PC synapse, pharmacological activation of mGlu4 receptors reversibly reduces excitatory synaptic transmission (Pekhletski et al., 1996; Lorez et al., 2003; Abitbol et al., 2008). Mechanisms underlying this phenomenon generally involve inhibition of presynaptic voltage-gated calcium channel (VGCC) activity and activation of presynaptic potassium channels, like inward rectifying K+ channels (GIRKs) (Niswender et al., 2008; Mercier and Lodge, 2014; Ribeiro et al., 2017). Consequently, action potential-evoked presynaptic calcium transients are diminished, leading to a decrease in vesicular glutamate release (Dittman and Regehr, 1996; Daniel and Crepel, 2001; Brown et al., 2003; Daniel et al., 2004; Abitbol et al., 2008). Along the same lines, extracellular adenosine, whose levels increase with hypoxic and ischemic events (Frengueilli et al., 2007; Melani et al., 2014) can have neuroprotective effects on the central nervous system through activation of presynaptic type 1 adenosine (A1) receptors (Dunwiddie and Fredholm, 1989; Dale et al., 2000; Dulla et al., 2005). Activation of these receptors can reduce excitatory synaptic transmission at central nervous system synapses for the duration of the insult (Gomes et al., 2011). This leads to an intriguing question: given the neuroprotective role attributed to mGlu4 and A1 receptors during an ischemic or hypoxic insult, are there functional interactions between these receptors, and if so, how do these interactions affect excitatory synaptic transmission at PF-PC synapses?

The cerebellar cortex provides an exemplary model for investigating the functional interplay between mGlu4 and A1 receptors, as both receptor types are expressed on granule cell PFs that innervate both PCs and molecular layer interneurons (Mateos et al., 1998; Corti et al., 2002; Wall et al., 2007; Klyuch et al., 2012; Zhang and Linden, 2012; Siddig et al., 2020). mGlu4 receptors are localized to the presynaptic active zone of PF terminals (Mateos et al., 1998; Corti et al., 2002; Siddig et al., 2020), positioned strategically to modulate action potential-driven synaptic transmission. A1 receptors are found on PF terminals (Rivkees et al., 1995; Dittman and Regehr, 1996), from which adenosine is released in an activity-dependent manner (Wall et al., 2007; Klyuch et al., 2012). Recent evidence from our laboratory has shown that in a model of simulated cerebellar ischemia, mGlu4 receptors reduce PF-PC synaptic transmission in the early stages of an excitotoxic insult (Bossi et al., 2018). Similarly, exogenous adenosine also reduces excitatory transmission at this same synapse (Kocsis et al., 1984; Blond et al., 1997).

Functional mGlu4 receptors exist exclusively as dimers (Conn and Pin, 1997) and both mGlu4 and A1 receptors can interact with other G protein-coupled receptors (GPCR), sometimes forming heterometric complexes (Nicoletti et al., 2023). To our knowledge, functional interactions between mGlu4 and A1 receptors in modulating glutamatergic synaptic transmission in the cerebellar cortex, have not been explored. We sought to address this by investigating whether mGlu4 receptor activity influences A1 receptor-mediated changes in excitatory transmission. Our approach combined whole-cell patch clamp recordings of PCs with presynaptic PF fluorometric calcium measurements in acute rat and mouse cerebellar cortical slices. Our findings suggest that there are functional interactions between these two receptor types at the PF-PC synapse, with potentially important consequences for receptor activation, function, and neuroprotection under pathophysiological conditions associated with elevated glutamate and adenosine levels.

## 2 Materials and methods

### 2.1 Animals

Animals were housed with *ad libitum* access to food and water at 22°C–23°C in a standard 12 h light–dark cycle. Animal care and euthanasia procedures were in accordance with European legislation (2010/63EU Council Directive Decree) and followed Annex IV of the French Decree (1 February 2013) establishing the guidelines for euthanasia. All efforts were made to minimize animal suffering and to reduce the number of animals used in this study. Sprague Dawley rats and C57BL/6 wild-type (WT) mice came from Janvier Laboratories (France). To generate mutant mice lacking the mGlu4 receptor, Grm4^+/−^ (B6.129-Grm4tm1Hpn/J) animals (Pekhletski et al., 1996) produced on a C57BL/6 background, were purchased from Jackson Laboratories (United States), with Charles River Laboratories (France) as the international import and distribution agent.

### 2.2 Slice preparation

21 to 34–day-old male Sprague-Dawley rats, male C57BL/6 mGlu4+/+ or male mGlu4^−/−^ mice were anesthetized with 2-bromo-2-chloro-1,1,1-trifluoroethane, then decapitated. Coronal or sagittal slices (250 µm) were cut from the cerebellar vermis in ice-cold oxygenated Bicarbonate Buffered Saline (BBS) (<1°C) with a vibratome (Microm HM 650 V). The BBS contained (in mM): NaCl, 138.6; KCl, 3; NaHCO_3_, 24; KH_2_PO_4_, 1.15; MgSO_4_, 1.15; CaCl_2_, 2; glucose, 10 and was gassed with 95% O_2_ and 5% CO_2_ (osmolarity, 330 mosmol/L; pH, 7.35). For electrophysiological recordings and calcium sensitive flurometric measurements, slices were transferred to a chamber on an upright microscope (Zeiss) and perfused at 2 mL per min with oxygenated BBS, supplemented with the GABA_A_ receptor antagonist, GABAzine (5 μM). All experiments were performed at 30°C–32°C. L-AP4 (L-(+)-2-amino-4-phosphonobutyric acid) and MSOP ((RS)-α-methylserine-O-phosphate), were purchased from Tocris Bioscience, GABAzine (SR95531) from Abcam and 2-CA (2-chloroadenosine) from Sigma-Aldrich.

### 2.3 Electrophysiology

Whole-cell patch-clamp recordings of PCs were performed in sagittal slices obtained from 15 Sprague-Dawley rats, 5 mGlu4 +/+ mice and 5 mGlu4 −/− mice. Recordings were made with an Axopatch-1D amplifier (Axon Instruments, United States) and PC somata were visualized using Nomarski optics and a ×63 water-immersion objective. Patch pipettes (3–3.5 MΩ) were filled the following solution (in mM): K-gluconate, 140; KCl 6; HEPES, 10; EGTA, 0.75; MgCl_2_, 1; Na-GTP, 0.4; Na_2_-ATP, 4; pH adjusted to 7.3 with KOH; 300 mosmol/L. PCs were voltage-clamped at −60 mV and junction potentials were corrected. PFs were stimulated every 6 s (0.17 Hz) through a glass saline-filled monopolar electrode placed in the molecular layer to evoke excitatory postsynaptic currents in PCs (PF-EPSCs). Recorded PF-EPSCs were filtered at 5 kHz, digitized on-line at 20 kHz, and analyzed on and off-line with Elphy (G. Sadoc, France), Igor (Wavemetrics, United States), and Clampfit (Axon Instruments, United States) software. Series resistance was partially compensated (60%–75%), as previously described by Llano et al. (1991). Recordings were terminated if this resistance increased by more than 20% of the initial value. PCs were clamped at −60 mV but PF-EPSCs were elicited on a 10 mV hyperpolarizing voltage step, which allowed monitoring of passive membrane properties (cell capacitance and membrane resistance). Cells were discarded from analysis if these parameters varied more than 20%. PF-EPSCs were evoked with pairs of stimuli of the same intensity (paired pulse stimulation), with an inter-stimulus interval of 40 ms. The paired-pulse ratio (PPR) of these responses was calculated on-line as EPSC2/EPSC1 amplitude (Atluri and Regehr, 1996).

### 2.4 Calcium sensitive fluorometric measurements

Using coronal slices obtained from 6 Sprague-Dawley rats and 6 mGlu4+/+ mice, presynaptic calcium transients were recorded by photometry as previously described (Daniel and Crepel, 2001). Briefly, the membrane-permeable calcium sensitive dye Fluo4-FF-AM (100 μM, Molecular Probes) was delivered into the molecular layer of the cerebellar cortex, where PFs contact PC dendrites, using a micropipette (30 min). After allowing the fluorochrome to diffuse within the fibers, a confined window (20 × 50 µm) of labeled PFs was illuminated at a single excitation wavelength (mercury Short Arc HBO, 480 ± 22 nm) gated with an electromechanical shutter (Uniblitz, Rochester, NY, United States). Labeled PFs were stimulated every 30 s with a single train of 5 electrical stimuli (100 Hz) delivered through a saline-filled monopolar electrode. Evoked Ca^2+^ sensitive changes in fluorescence were acquired through a ×63 water-immersion objective of an epifluorescence microscope (Zeiss, LePeck France), with a barrier filter at 530 ± 30 nm and converted to an electric signal by a photometer (Nikon). Fluorometric measurements were analyzed on- and off-line using Elphy software. Data, corrected for dye bleaching, were expressed as relative fluorescence changes ΔF/F, where F is the baseline fluorescence intensity, and ΔF is the change induced by PF stimulation in real time. Each recording was digitalized and analyzed individually using Microsoft Excel software.

### 2.5 Data statistical analysis

To analyze the effects of L-AP4 and 2-CA (alone or in the presence of MSOP) on PF–EPSCs, the amplitude of evoked currents was calculated and normalized to control values. Control amplitude and PPR was that measured before any drugs were applied. The average change in PF-EPSC amplitude and PPR associated with the L-AP4 (or 2-CA) effect was calculated over a 5-minute period at peak effect of the drug. All data are expressed as mean ± S.E.M. Statistical significance was assessed for normally distributed data using a paired or unpaired Student’s t test or analysis of variance (ANOVA) with *p* < 0.05 (two-tailed) considered as significant. The similarity of variances between each group of results was tested using Ficher’s test with α = 0.02. “n” indicates the number of cells included in the statistics. A *p*-value <0.05 was considered statistically significant.

## 3 Results

### 3.1 Activation of presynaptic mGlu4 and A1 receptors diminishes excitatory transmission at PF-PC synapses

**FIGURE 1:**
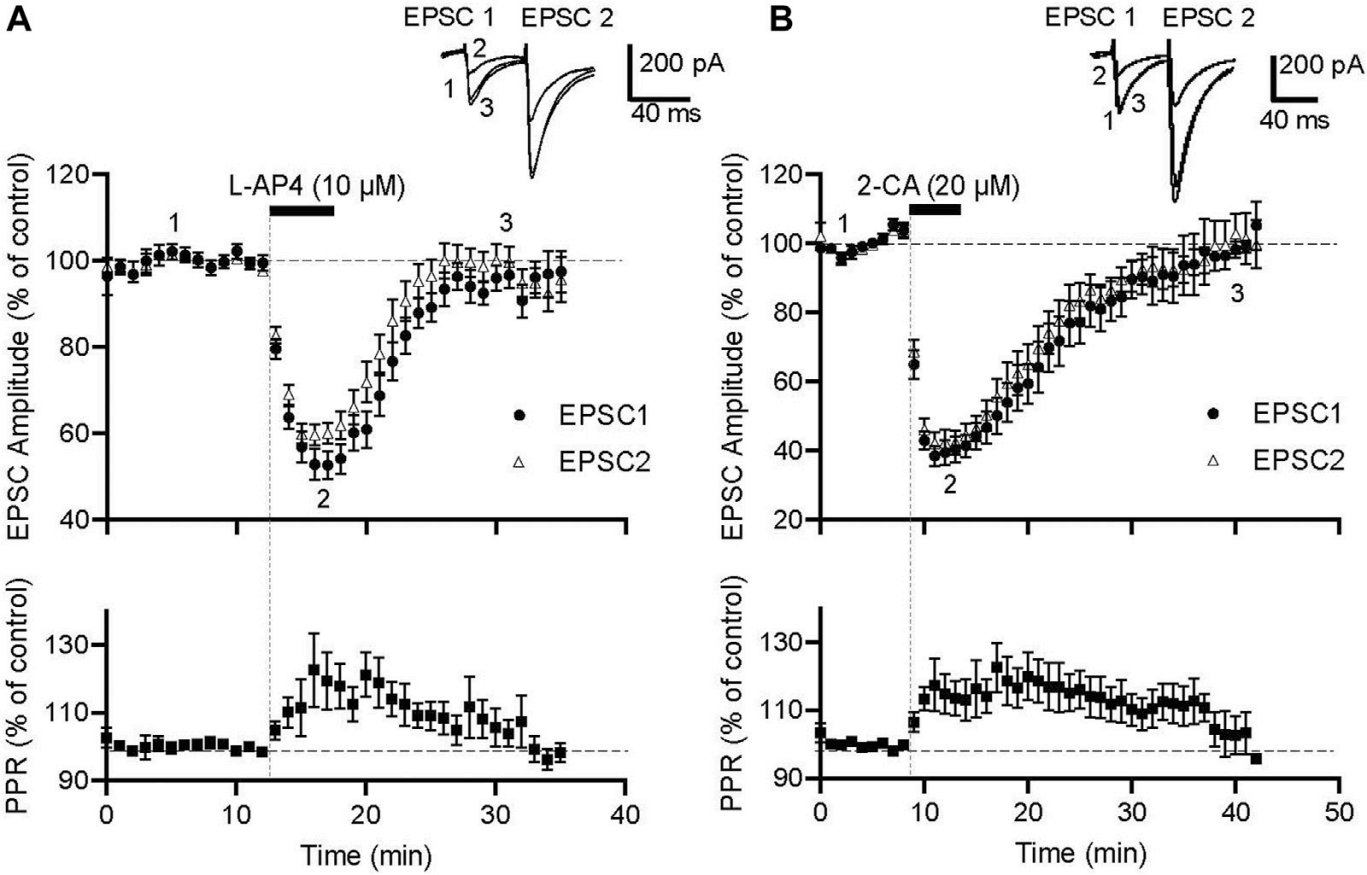
Pharmacological activation of mGlu4 and A1 receptors reduces evoked PF-EPSC amplitude in *p*Cs in rat. **(A)** Normalized amplitude of the first (filled circles) and second (open triangles) PF-evoked EPSCs before, during and after 5-min applications of the group III mGluR agonist, L-AP4 (10 μM). Upper traces are averages of 5 consecutive evoked responses from one experiment recorded before (1), at the peak of the L-AP4 effect (2) and during recovery (3). The PPR, calculated as EPSC2/EPSC1 amplitude and normalized to control values, shows transient increases during L-AP4 response (n = 16). **(B)** Normalized amplitude of the first (filled circles) and second (open triangles) PF- evoked EPSCs before, during and after 5-min applications of the adenosine type 1 receptor agonist, 2-CA (20 μM). Upper traces are averages of 5 consecutive evoked responses from one experiment recorded before (1), at the peak of the 2-CA effect (2) and during recovery (3). Normalized values of PPR show transient increases during the 2-CA response (n = 10).

Before investigating functional interactions between mGlu4 and A1 receptors, we documented the effects of pharmacological activation of each of these receptor types on PF-PC evoked excitatory synaptic transmission in sagittal slices of rat cerebellar cortex. As illustrated in the inserts in Figure 1, paired pulse stimulation evoked EPSCs showcasing significant paired-pulse facilitation (PPF). PPF is a form of presynaptic short-term plasticity characteristic of synapses with low transmitter release probability (Atluri and Regehr, 1996). mGlu4 receptors were activated pharmacologically with near saturating concentrations of L-AP4 (10 μM, 5-min), a group III mGluR agonist. While L-AP4 is non-specific to all group III mGlu, mGlu4 is the only subtype functional at the PF-PC synapse and 5 min applications of L-AP4 produced maximal depressive effects (Abitbol et al., 2008). A1 receptors were activated with saturating concentrations of 2-CA (20 μM, 5-min), an adenosine analogue, commonly used as an agonist to activate these presynaptic receptors (Dittman and Regehr, 1996).

Figure 1A shows that activation of mGlu4 receptors with bath application of L-AP4 reversibly reduced the amplitude of both the first and second evoked EPSC. EPSC-1 amplitude was reduced by 44.5% ± 3.0% compared to control (pre-L-AP4) values while EPSC-2 amplitude was reduced by 37.8% ± 2.6% (n = 16, *p* < 0.0001). This corresponded to a significant increase in the PPR to 117% ± 7% of pre-L-AP4 values (*p* = 0.0279), coherent with a presynaptic locus for these mGlu4 receptors. Figure 1B shows that activation of A1 receptors also reduced evoked excitatory synaptic transmission at the PF-PC synapse. At the peak effect of 2-CA application, EPSC-1 amplitude was reduced by 61.4% ± 3.4% compared to control (pre-2-CA) values while EPSC-2 amplitude was reduced by 57.8% ± 3.4% (n = 10, *p* < 0.0001). The 2-CA effect was accompanied by an increase in the PPR to 115.1± 5.5% of control (pre-2-CA) values (*p* = 0.0222), confirming the involvement of presynaptic A1 receptors. These results affirm that pharmacological activation of mGlu4 or A1 receptors reversibly and significantly reduces excitatory synaptic transmission at the PF-PC synapse. Furthermore, the observed increases in PPR during agonist application align with a presynaptic origin for these effects. For the sake of clarity, subsequent figures illustrating electrophysiology experiments will only show data for the first EPSC (EPSC-1) and the PPR.

### 3.2 Sequential activation of mGlu4 and A1 receptors partially occluded evoked presynaptic calcium transients in PF terminals

It has been reported, including by our own laboratory, that presynaptic mGlu4 and A1 receptors decrease excitatory synaptic transmission at the PC-PF synapse by diminishing calcium ion influx through presynaptic VGCCs (Dittman and Regehr, 1996; Abitbol et al., 2012). Using fluorometric measurements of evoked calcium transients in coronal sections of rat cerebellar cortex, we looked for functional interactions between mGlu4 and A1 receptor types at presynaptic PF terminals. We tested whether pharmacological activation of mGlu4 receptors occluded or reduced the effect of subsequent activation of A1 receptors, and *vice versa*. This experimental paradigm can indicate whether different receptor types interact functionally and/or share common signaling pathways initiating cellular responses. If mGlu4 and A1 receptors act independently at this synapse, sequential activation of these receptors would be expected to have additive effects on the amplitude of evoked calcium transients. Data were quantified as the change in evoked fluorescence (ΔF/F): first at the peak effect of the first agonist compared to control (before agonist application) and then at the peak effect of the second agonist in the presence of the first. The second agonist was applied during the peak effect of the first agonist.

**FIGURE 2:**
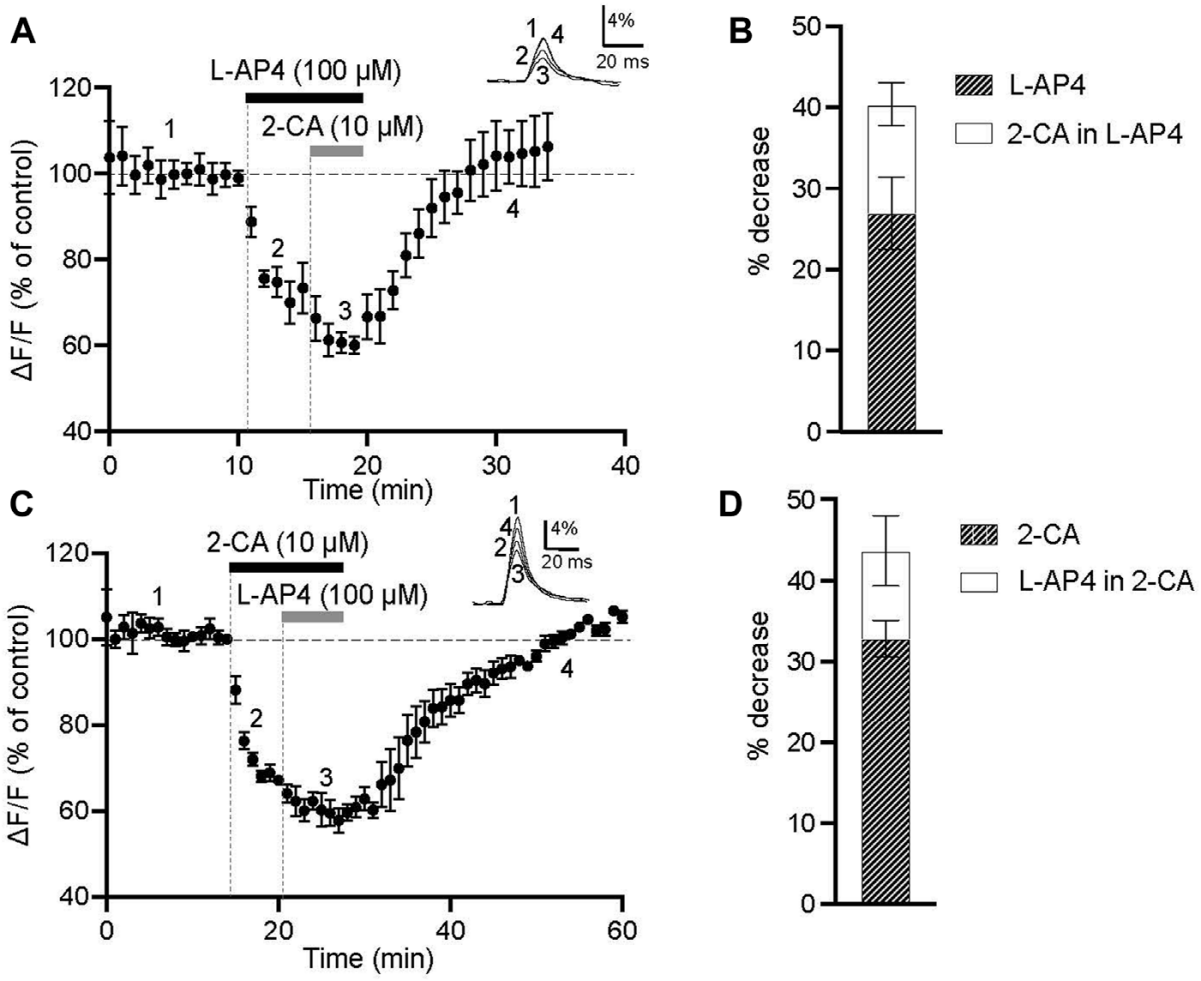
Sequential activation of mGlu4 and A1 receptors show partial occlusion in reducing evoked presynaptic calcium transients in PF terminals in rat. **(A)** Normalized amplitude of peak fluorescence transients (ΔF/F) evoked by 5 PF stimulations (delivered at 100 Hz) before agonist application (1), during 5-min application of 100 μM L-AP4 alone (2), during 5-min application of 10 μM 2-CA in the presence of L-AP4 (3), and during recovery (4) (n = 5). Upper traces are averages of 5 consecutive evoked responses from one experiment recorded before (1), at the peak of the L-AP4 effect (2), at the peak of the 2-CA effect in L-AP4 (3) and in recovery (4). **(B)** The stacked bar graph shows the percent decrease of evoked fluorescence transients at maximal effect of L-AP4 alone (hatched bar) and the 2-CA effect in the presence of L-AP4 (white bar). **(C)** Normalized amplitude of peak fluorescence transients (ΔF/F) evoked by 5 PF stimulations (delivered at 100 Hz) before agonist application (1), during 5-min application of 10 μM 2-CA alone (2), during 5-min application of 100 μM L-AP4 in the presence of 2-CA (3), and during recovery (4) (n = 5). Upper traces are averages of 5 consecutive evoked responses from one experiment recorded before (1), at the peak of the 2-CA effect (2), at the peak of the L-AP4 effect in 2-CA (3) and in recovery (4). **(D)** The stacked bar graph shows the percent decrease of evoked fluorescence transients at maximal effect of 2-CA alone (hatched bar) and the L-AP4 effect in the presence of 2-CA (white bar). All plots illustrate the mean ± SEM.

As shown in Figures 2A,B, bath application of a saturating concentration of L-AP4 (100 μM, 5-min) reduced the amplitude of evoked presynaptic calcium transients by 27% ± 4.5% compared to control values (n = 5, *p* = 0.0038). This decrease is coherent with our previously published data in which we showed that 5 min application of 100 μM L-AP4 alone depressed evoked presynaptic calcium influx by 25.3% ± 2.3% (Abitbol et al., 2012). Subsequent application of 2-CA (10 μM, 5 min) in the presence of L-AP4 further decreased this amplitude by 13.4% ± 2.7% compared to that measured in L-AP4 alone (*p* = 0.0314). In a second series of experiments illustrated in Figures 2C,D, we reversed the order of agonist application (same concentrations and duration). Bath application of 2-CA reduced evoked calcium transient amplitude by 31.8% ± 2.4% (n = 4, *p* < 0.0001). In preliminary experiments, we found that the same application of 2-CA reversibly decreased evoked presynaptic calcium transients by 28.4% ± 4.2% (n = 8, Supplementary Figure S1). Subsequent application of L-AP4 in the presence of 2-CA further decreased this amplitude by 12.7% ± 5.1% compared to that measured in 2-CA alone (*p* = 0.0021). These data, represented as a stacked bar graph in Figures 2B,D, clearly show that L-AP4 and 2-CA exert more potent depressive effects on evoked PF calcium transients when applied individually than when applied sequentially. If mGlu4 and A1 receptors act independently at this synapse, one would anticipate that sequential activation of these receptors would have additive effects on the decreases in amplitude of evoked calcium transients. In other words, decreases in evoked transient amplitude in L-AP4 alone (hatched bar Figure 2B), and in the presence of 2-CA (white bar Figure 2D) would be similar. Our results hint at potential functional interactions between these two presynaptic receptors. It would have been interesting to assess the effect of sequential applications of mGlu4 and A1 receptor agonists on synaptic transmission by recording evoked EPSCs from PCs under these conditions.

### 3.3 Pharmacological blockade of mGlu4 receptors enhances A1 receptor–mediated decreases in PF-PC excitatory transmission

**FIGURE 3:**
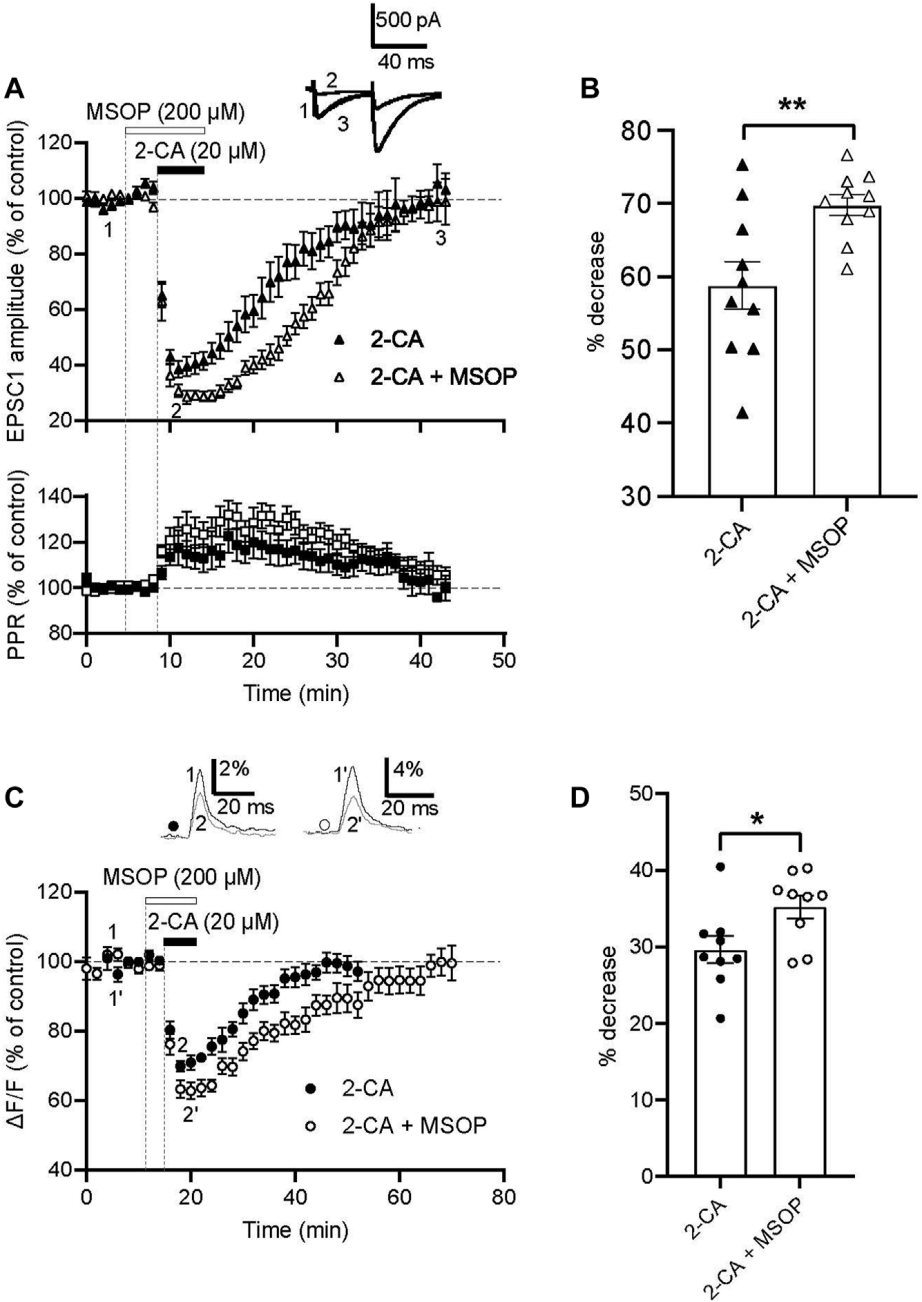
Blocking mGlu4 receptors potentiates the depressive effect of A1 receptor activation on PF evoked EPSCs and presynaptic calcium transients in rat. **(A)** Normalized amplitude of EPSC-1 before, during and after 20 μM 2-CA applied alone (closed triangles, n = 10, same data as in Figure 1B) and in the presence of 200 μM MSOP (open triangles, n = 10). Normalized values of PPR show transient increases during 2-CA–mediated depression of evoked EPSC amplitude when administered alone (black squares) or in the presence of MSOP (white squares). **(B)** Percent decrease in EPSC-1 amplitude at the peak effect of 2-CA alone (filled triangles) and when applied in the presence of MSOP (open triangles). Plots are the mean ± SEM, *p* = 0.0185. **(C)** Normalized amplitudes of peak fluorescence transients (ΔF/F) evoked by 5 PF stimulations (delivered at 100 Hz) before, during and after 2-CA (20 μM) applied alone (closed circles, n = 9) and in the presence of MSOP (200 μM, open circles, n = 9). Upper traces are averages of 5 consecutive evoked responses from one experiment recorded before and at the peak of the 2-CA effect when 2-CA is applied alone (1, 2) and in the presence of MSOP (1′, 2′). **(D)** Percent decrease in evoked fluorescence transients at maximal 2-CA effect alone (filled circles) and in the presence of MSOP (open circles). Plots are mean ± SEM, *p* = 0.0287.

We next explored whether blocking mGlu4 receptor activity could affect A1 receptor-mediated reductions in excitatory synaptic transmission at the PF-PC synapse. We compared the amplitude of evoked EPSCs and their PPR under two experimental conditions: bath application of 2-CA alone (20 μM, 5-min, n = 10) and in the presence of the group III mGlu receptor antagonist, MSOP (200 μM, 10-min, n = 10). As shown in Figure 3A, bath application of MSOP alone had no effect on EPSC-1 amplitude (98.3% ± 1.5% before MSOP and 101.2% ± 0.6% in MSOP, *p* = 0.1443), and the PPR remained unchanged (98.6% ± 0.9% before MSOP and 100.3 ± 0.4 in MSOP, *p* = 0.1239). In the presence of MSOP, 2-CA significantly decreased the amplitude of evoked EPSCs and concomitantly increased the PPR of PF-PC synaptic responses. However, while MSOP resulted in significantly larger 2-CA–mediated decreases in EPSC amplitude as shown in the bar graph in Figure 3B (*p* = 0.0185), the change in PPR with 2-CA in MSOP was not different to that obtained in 2-CA alone (*p* = 0.2799). Close inspection of Figure 3A reveals that in the presence of MSOP, the depressive effect of 2-CA on evoked EPSC-1 amplitude was not only larger, it also lasted longer than when 2-CA was applied alone.

We confirmed these mGlu4 and A1 receptor interactions affect presynaptic calcium influx by using fluorometric measurements of evoked PF calcium transients in coronal sections of rat cerebellar cortex. Figure 3C shows that 2-CA (10 μM, 5-min) decreased evoked calcium transients and in the presence of MSOP (200 μM, 10-minutes), this effect was enhanced. In addition, the duration of the 2-CA effect was longer in the presence of MSOP. These data are quantified in the bar graph shown in Figure 3D. 2-CA alone decreased ΔF/F by 29.6% ± 1.8% compared to control values and in the presence of MSOP the average decrease rose significantly to 35.2% ± 1.5% (*p* = 0.0287, n = 9).

### 3.4 A1 receptor–mediated decreases in PF-PC synaptic transmission are similar in mouse cerebellar cortex when mGlu4 receptors are blocked pharmacologically or deleted genetically

**FIGURE 4:**
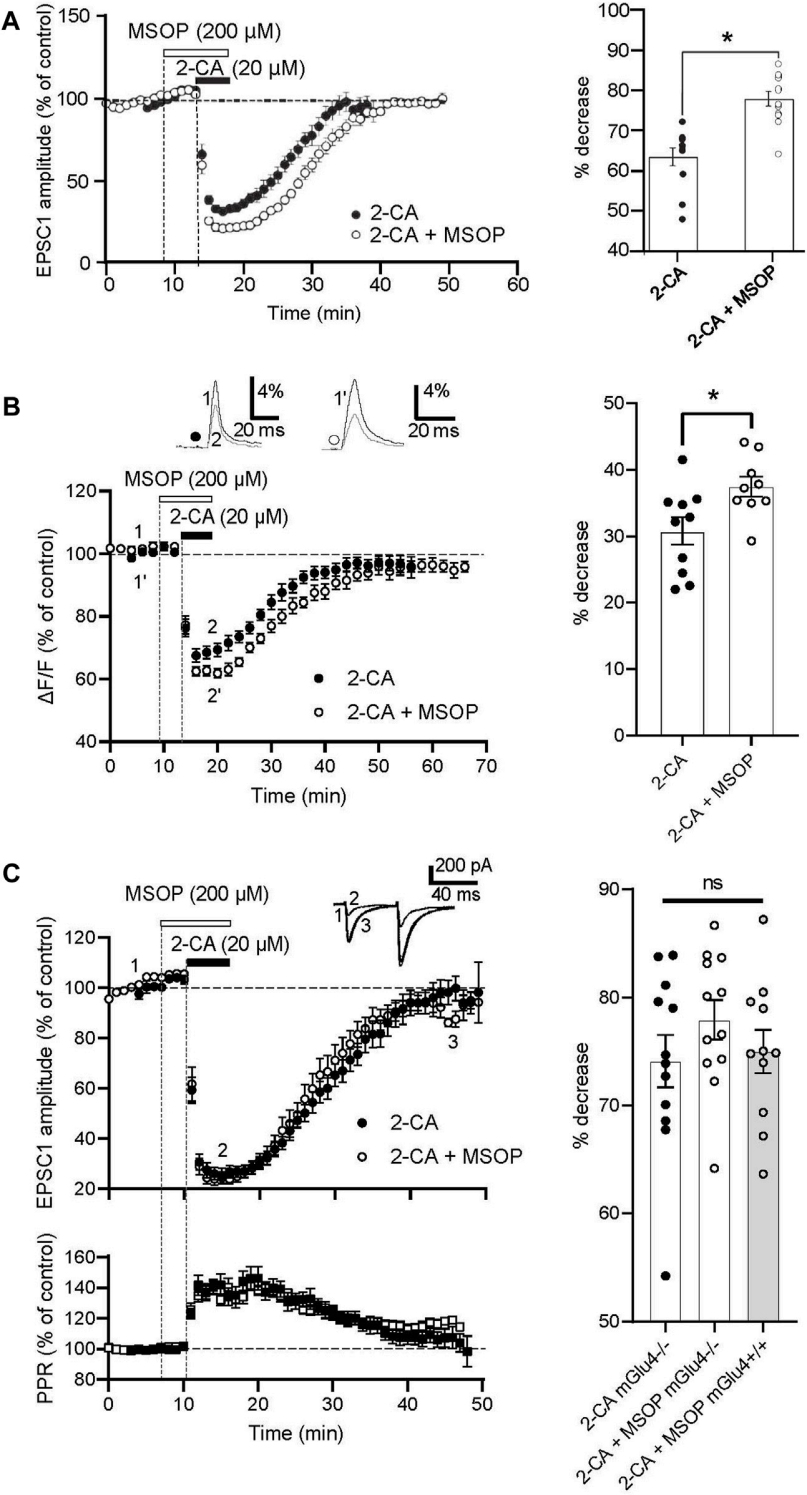
Pharmacological inactivation of mGlu4 receptors potentiates the depressive effect of A1 receptor activation in mGlu4+/+ mice but not in mGlu4−/− mice. **(A)** Normalized amplitude of the EPSC-1 before, during and after 2-CA application (20 μM) alone (closed circles, n = 11) and in the presence of MSOP (200 μM, open circles) (n = 12) in mGlu4+/+ slices. The bar graph shows the percent decrease in EPSC-1 amplitude with 2-CA alone (filled circles) and 2-CA + MSOP (open circles). Plots are mean ± SEM, *p < 0.05*. **(B)** Normalized amplitudes of peak fluorescence transients (ΔF/F) evoked by 5 PF stimulations (delivered at 100 Hz) before, during and after 2-CA (20 μM) applied alone (closed circles, n = 9) and in the presence of MSOP (200 μM, open circles, n = 9) in mGlu4+/+ slices. Upper traces are averages of 5 consecutive evoked responses from one experiment recorded before and at the peak of the 2-CA effect when 2-CA is applied alone (1, 2) and in the presence of MSOP (1′, 2′). The bar graph shows percent decrease in evoked fluorescence transients at maximal 2-CA effect alone (filled circles) and in the presence of MSOP (open circles). Plots are mean ± SEM, *p* = 0.0201. **(C)** Normalized amplitude of the EPSC-1 before, during and after 2-CA application (20 μM) alone in mGlu4−/− slices (closed circles, n = 11) and in the presence of MSOP (200 μM, open circles) (n = 10). Upper traces are averages of 5 consecutive evoked responses from one experiment recorded before (1), at the peak of the 2-CA effect in MSOP (2) and during recovery (3). Normalized values of PPR show transient increases during 2-CA–mediated depression of evoked EPSC amplitude when administered alone (black squares) or in the presence of MSOP (white squares). Percent decrease of EPSC-1 amplitude in mGlu4−/− slices with 2-CA alone (filled circles), in the presence of MSOP (open circles) and in mGlu4+/+ slices in the presence of MSOP (open circles, grey bar). Plots are mean ± SEM.

We then extended our results to the mouse PF-PC synapse to benefit from animals devoid of mGlu4 receptors (see Pekhletski et al., 1996). Figure 4A shows that like for rats, in wild type mice, 2-CA (10 μM, 5-min) decreased evoked EPSC amplitude and that this decrease was significantly enhanced in the presence of MSOP (200 μM, 10-min). In addition, the duration of the 2-CA effect was longer in the presence of MSOP. The effect of 2-CA on evoked EPSC amplitude was similar in both rat and wild type mouse slices (Supplementary Figure S2). Fluorometric measurements of evoked PF calcium transients in wild type mice illustrated in Figure 4B show that 2-CA alone decreased ΔF/F by 30.8% ± 2% compared to control values and in the presence of MSOP the average decrease rose significantly to 37.5% ± 1.5% (*p* = 0.0201, n = 10). It appears that antagonizing mGlu4 receptors enhances subsequent A1-mediated depressive effects on evoked excitatory transmission at this synapse in both rats (Figure 3) and wild type mice (Figures 4A, B), and involves presynaptic mechanisms.

Finally, to further our hypothesis that mGlu4 receptor activity influences A1 receptor–mediated decreases in excitatory synaptic transmission at the PC-PF synapse, we compared the effects of A1 receptor activation in the presence of MSOP in slices obtained from wild-type mice (mGlu4+/+) with those obtained from mGlu4 knock-out mice (mGlu4−/−). As shown in Figure 4C, in mGlu4−/− slices, bath application of 2-CA alone (20 μM, 5-min) reversibly reduced EPSC-1 amplitude by 73.2% ± 2.5%. This depression resembled that observed when 2-CA was applied in the presence of MSOP (200 μM, 10-min). As expected, changes in the PPR following 2-CA application were similar in these two conditions (24.2% ± 5.9% for 2-CA in MSOP, n = 10, and 37.4% ± 4.5% for 2-CA alone in mGlu4−/− slices, n = 11, *p* = 0.0866). In mGlu4−/− slices, the percentage of depression in evoked EPSC-1 amplitude with 2-CA application was not different to that observed with co-application of 2-CA and MSOP (*p* = 0.7791). Furthermore, the depression in evoked EPSC amplitude with 2-CA applied in the presence of MSOP was similar in mGlu4−/− and mGlu4+/+ mice (*p* = 0.2910, n = 12). These data are summarized in the bar graph. Taken together these results indicate that inhibiting (or knocking out) presynaptic mGlu4 receptors potentiates the depressant effect of presynaptic A1 receptor activation on glutamate transmission at the PF-PC synapse thus supporting our hypothesis of functional interactions between these two receptor types at PF terminals.

## 4 Discussion

Our results shed light on functional interactions between mGlu4 and A1 receptors in the cerebellar cortex. Both these receptor types, situated on PF terminals, can play a neuroprotective role by modulating glutamate release at the PF-PC synapse (Dittman and Regehr, 1996; Lorez et al., 2003; Mercier and Lodge, 2014; Bossi et al., 2018). Since it is likely that both mGlu4 and A1 are activated during pathological situations such as epileptic seizures or ischemic insults (Dunwiddie et al., 2001; Dale and Frenguelli, 2009; Melani et al., 2014), if and how the activation of one receptor type influences the other is of interest, particularly from a therapeutic perspective.

Under our experimental conditions, and in agreement with our previous studies (Abitbol et al., 2008; Abitbol et al., 2012), mGlu4 receptors do not appear to be active under “basal” physiological conditions since blocking these receptors (with MSOP) had no effect on PF evoked EPSCs or presynaptic calcium transients. However, mGlu4 receptor blockade potentiated A1 receptor–mediated decreases in both evoked PF calcium transients and excitatory transmission in both rats and wild type mice. This was not observed in mGlu4 −/− slices. Furthermore, when mGlu4 and A1 receptors were activated sequentially, the resulting decrease in evoked calcium transient amplitude was far less than the addition of the two independent depressive effects. If mGlu4 and A1 receptors act independently at this synapse, sequential activation of these receptors would be expected to have additive effects on the amplitude of evoked calcium transients. Our findings suggest possible functional interactions between these two receptor types.

What could be the nature of this interaction? While many mGluRs form heterometric complexes, dimerizing with other types of GPCRs (Lee et al., 2020; McCullock and Kammermeier, 2021; Nicoletti et al., 2023), to our knowledge, heterodimerization or even functional interactions *via* intracellular signaling proteins between mGlu4 and A1 receptors have not been demonstrated. Being said, different types of GPCRs may interact through β and γ subunits of G proteins. For example, Smrcka and Fisher (2019) have demonstrated that receptors coupled to G_i/o_ proteins may amplify the activity of receptors coupled to G_s_ or G_q/11_ proteins because Gβγ subunits can activate certain isoforms of adenylate cyclase or phospholipase-CB binding domains. An example of this is found in Purkinje cells where the Gβγ subunits produced by the activation of the G_i_-coupled GABA_B_ receptor enhance G_q_-coupled mGlu1 responses (Rives et al., 2009). These interactions have significant implications for downstream signaling pathways and the outcome of receptor activation (Nicoletti et al., 2023).

Another possible source of interaction between mGlu4 and A1 receptors could be through actors within a common or converging signaling pathway. mGlu4 receptors, like other group III mGluRs, have been repeatedly reported to be negatively coupled to the adenylate cyclase signaling pathway via pertussis toxin sensitive G_i/o_ proteins (Conn and Pin, 1997). While we have previously demonstrated that the vast majority of mGlu4 depressive effects on glutamate transmission at the PF-PC synapse involve the inhibition of voltage-gated calcium channels principally by way of a non-canonical, pertussis toxin insensitive intracellular cascade involving activation the PLC-PKC signalling pathway, it appears that other G-proteins, such as Gi could also be involved, *albiet* to a lesser extent (Abitbol et al., 2012). Indeed, proximity ligation assays show that in the molecular layer of the cerebellar cortex, mGlu4 receptors are in close proximity to both Gαq and Gαi/o proteins (Chardonnet et al., 2017). Given that A1 receptors are coupled to Gi/o proteins (Ciruela et al., 2010; Effendi et al., 2020), these two receptors may interact somewhere within the adenylate cyclase signalling pathway. However, given the small degree of mGlu4 signaling that persisted after inhibition of the PLC-PKC pathway (Abitbol et al., 2012), this probably cannot explain the significant increases in 2-CA mediated depression of presynaptic calcium transients and evoked EPSCs that we see in the present study when mGlu4 receptors are antagonized with MSOP. Future experiments with sequential or co-application of mGlu4 and A1 agonists in the presence of a protein Gq inhibitor could help resolve this question.

Finally, mGlu4 and A1 presynaptic receptors could interact independently of interactions between their G-proteins. Our previous study employing peptide affinity chromatography demonstrates that native mGlu4 receptors interact with presynaptic vesicular trafficking proteins such as Munc18-1, SNAP-25, synapsin and syntaxin (Ramos et al., 2012). Interestingly, Nakajima et al. (2009) have reported that in cultured superior cervical ganglion cells transfected with relevant mGlu4 receptor residues, mGlu4 interacts with Munc-18 to inhibit vesicular release, and this independently of protein G activation. While we know of no studies linking A1 receptors to vesicular trafficking proteins, there is evidence for interactions with heat shock (stress) proteins that, among their many roles, may also modulate ligand binding and adenosine receptor signaling (Ciruela et al., 2010). Both mGlu4 and A1 receptors can interact with other receptor types. For example, at the PF-PC synapse, mGlu4 and GABA_A_ receptors are found on PF terminals but have opposite effects on glutamate release. Presynaptic GABA_A_ receptors, activated by GABA spill-over, increase glutamate release from PFs (Stell et al., 2007; Pugh and Jarr, 2011) while mGlu4 activation decreases it. Using cerebellar synaptosomes, Antflick and Hampson (2012) showed that mGlu4 and GABA_A_ receptors co-localise and interact functionally at PF terminals, but in a very surprising manner. Application of the mGlu4 agonist, L-AP4, in fact facilitates GABA_A_ receptor–dependent release of glutamate from PF terminals. This effect was abolished when synaptosomes were treated with pertussis toxin, indicating a role for G-proteins (linked to mGlu4 receptors) in this interaction. The authors suggest that among several possible hypothesis, mGlu4 could have ligand-independent but calcium-dependent effects on vesicular trafficking proteins, as proposed by Nakajima et al. (2009). Kamibubo et al. (2013) have revealed a fascinating interplay between postsynaptic mGlu1 and A1 receptors, which closely coexist on the cell surface of Purkinje cells and have the capacity to create heteromeric complexes. In these neurons the activation of A1 receptors prompted changes in the ligand affinity of mGlu1 receptors by influencing their structural conformation thus facilitating mGlu1responses. Interestingly, in this same cell type, Tabata et al. (2007) showed inhibitory interactions between these two receptors that were independent of protein G activation. More recently Taketo (2023) demonstrated that interactions between mGlu1 and A1 receptors promote increases in intracellular calcium concentrations in hippocampal Cajal-Retzius cells with consequences on radial migration of neurons in the developing hippocampus.

What could be the significance of mGlu4-A1 receptor interactions for cerebellar function in pathological conditions associated with increases in both adenosine and glutamate in and around the synaptic space? Adenosine plays a neuroprotective role during ischemia (Melani et al., 2014), in part by activating presynaptic A1 receptors that curtail glutamate release from their terminals (Frengueilli et al., 2007; Wall et al., 2007; Gomes et al., 2011). Likewise, mGlu4 receptors also have a protective role in conditions of elevated synaptic glutamate (Lorez et al., 2003; Dale and Frenguelli, 2009; Domin et al., 2016; Domin, 2022). In a model of oxygen-glucose deprivation, we recently showed that mGlu4 receptors are activated during early stages of simulated cerebellar ischemia, thus reducing glutamate release and excitatory transmission at the PF-PC synapse (Bossi et al., 2018). How could interactions between mGlu4 and A1 receptors protect brain tissue from an ischemic insult? Ischemia begins with a loss of blood flow, and with it a deficit in oxygen delivery and fundamental metabolites such as glucose. This leads to loss in ATP synthesis, dysregulation of energy dependent processes such as the maintenance of ionic gradients, and dramatic depolarization of neurons and astrocytes, resulting in release of even more glutamate, adenosine and many other neuromodulators (Rossi et al., 2007). During the initial phases of an ischemic insult, mGlu4 receptors mitigate excitotoxicity by reducing glutamate release at their own synapse. However, if the ischemia persists, these receptors may desensitize. GPCR desensitization can arise from different events that are not mutually exclusive: receptor phosphorylation, internalization and interactions with specific regulatory proteins such as arrestins. In cultured cerebellar granule cells and transfected HEK293 cells, mGlu4 receptor-dependent cAMP inhibition is not desensitized in response to agonist stimulation (Iacovelli et al., 2004). However, when HEK293 cells co-expressed a chimeric G_αq/o_ protein that redirected mGlu4 coupling from adenylate cyclase to phospholipase C, these receptors indeed desensitized (Mathiesen and Ramirez, 2006). As mentioned above, PF mGlu4 receptors act mostly through a G_αq_-PLC signaling pathway (Abitbol et al., 2012; Chardonnet et al., 2017). Any ensuing conformational change in mGlu4 may permit potentiation of concomitantly activated A1 receptors via intracellular protein-protein interactions, thus potentiating their modulatory effects on synaptic transmission. However, with severe ischemic insults, the neuroprotective effects of mGlu4 and A1 receptor activation will be lost in the cascade of cellular responses and may only be important during very early stages of the insult.

The interplay between type 1 adenosine and mGlu4 receptors underscores the complexity of receptor interactions. Further exploration of the mechanisms and functional implications of these interactions promises to expand our understanding of neurobiological processes, with important therapeutic potential.

## Data Availability

The raw data supporting the conclusions of this article will be made available by the authors, without undue reservation.

## In memoriam

This article is dedicated to the memory of HD who passed away on 22/11/2019.

## Supplementary material

SUPPLEMENTARY FIGURE S1 Effect of Adenosine and 2-CA on evoked calcium transients in rat PF terminals. **(A)** Normalized amplitudes of peak fluorescence transients (ΔF/F) evoked by PF before, during and after bath application of 100 μM adenosine (n=10). **(B)** Normalized amplitudes of peak fluorescence transients (ΔF/F) evoked by PF before, during and after bath application of 10 μM 2-CA (n=8). **(C)** Percent decrease in evoked calcium transients at maximal adenosine and 2-CA effects. Bar graphs show mean ± SEM.

SUPPLEMENTARY FIGURE S2 Effect of A1 receptor activation on evoked EPSC amplitude in rat and mGlu+/+ mice. Normalized amplitude of the EPSC-1 before, during and after 5 min 2-CA application (20 μM) alone in rat (closed circles, n = 10) and mGlu+/+ mice (open circles, n = 11) slices. Normalized values of PPR show transient increases during 2-CA – mediated depression of evoked EPSC amplitude in both rat and mouse slices. Plots are mean ± SEM. **(B)** The bar graph shows the percent decrease in EPSC-1 amplitude with 2-CA application in rat (filled circles) and mGlu+/+ (open circles) slices. Plots are mean ± SEM. There is no difference between these two groups.
