# Supplementary figures and images for: Interplay between metabotropic glutamate type 4 and adenosine type 1 receptors modulate synaptic transmission in the cerebellar cortex
Source: Front Pharmacol. 2024 Aug 15;15:1406238. doi: 10.3389/fphar.2024.1406238 (PMC11358600; doi:10.3389/fphar.2024.1406238)

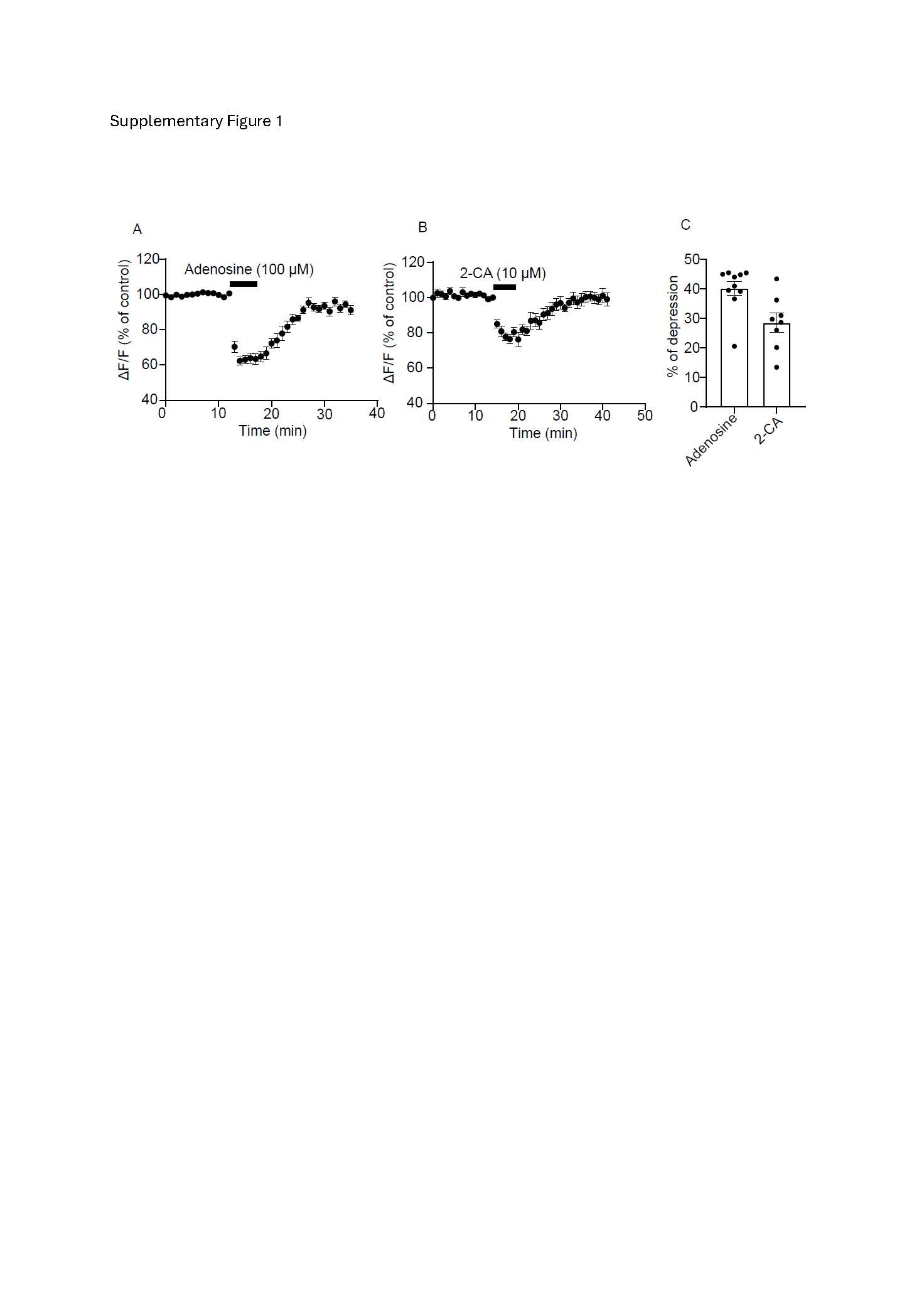

Supplement: Supplementary file 1 [file Image1.JPEG]

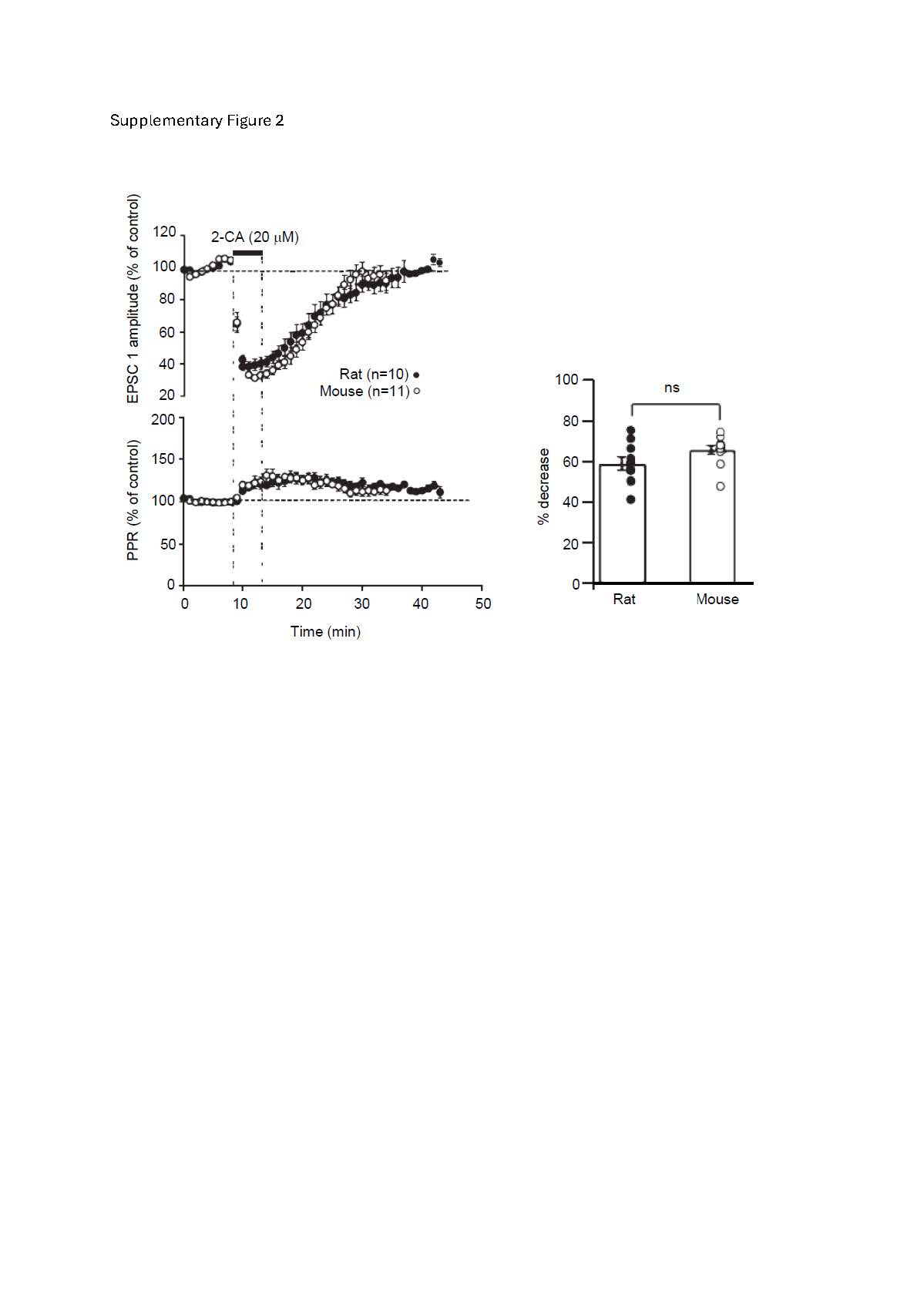

Supplement: Supplementary file 2 [file Image2.JPEG]
